# Enzymatic transesterification of palm stearin and olein blends to produce zero-trans margarine fat

**DOI:** 10.1186/1472-6750-12-48

**Published:** 2012-08-13

**Authors:** Mohamed Sellami, Hanen Ghamgui, Fakher Frikha, Youssef Gargouri, Nabil Miled

**Affiliations:** 1 Laboratoire de Biochimie et de Génie Enzymatique des Lipases, ENIS, Université de Sfax, route de Soukra, BPW 3038-1173, Sfax, Tunisia

## Abstract

**Background:**

Food industries aim to replace trans fat in their products by formulations having equivalent functionality and economic viability. Enzymatic transesterification can be a technological option to produce trans free fats targeting commercial applications.

**Results:**

Palm stearin and palm olein blends in different ratios were enzymatically transesterified in a solvent free system using a *Rhizopus oryzae* lipase immobilised onto CaCO_3_ to produce a suitable fat for margarine formulation. Slip melting points and triacylglycerols profiles were evaluated upon transesterification. Results indicated that all transesterified blends had lower slip melting points than their non transesterified counterparts. Furthermore, the triacylglycerols profile showed a decrease in the concentration of the high melting point triacylglycerols. The rheological analysis showed that margarine prepared with the transesterified blend showed a better spreadability than that of a control margarine prepared with non transesterified fat. Adding powder of dry bark orange to margarine preparation improved its colour and fairly affected its spreadability and rheological behaviour. The margarine prepared with transesterified fat displayed a rheological behaviour that was comparable to that of commercial sample.

**Conclusions:**

This study is an ecofriendly approach to the utilization of relatively low value bioresources like palm stearin and palm olein for making margarine free of trans fatty acids that are now implicated as risk factor for heart diseases.

## Background

Margarine was originally developed in 1869 as an alternative to butter which was in short supply and was also expensive [1]. Margarine is a water-in-oil emulsion. The aqueous phase consists of water, salt and preservatives. The fatty phase, which contributes to the polymorphic behaviour of margarine, is a blend of oils and fats, antioxidants and emulsifiers. Traditionally, the solid fat content of margarine is obtained by hydrogenation of liquid oils. Hydrogenation results in the formation of trans fatty acids where some cis double bonds are rearranged to trans bonds [2,3]. Several studies have suggested a direct relationship between trans fatty acids and increased risk for coronary heart diseases as well as raise of plasmatic lipid levels [3-7]. Different processes are currently available for the production of zero-trans solid fats in the food industry including chemical [8] or enzymatic transesterification [9,10]. Chemical transesterification usually needs cleaning process to remove the residual catalyst besides the formation of undesirable products. It is being successfully replaced by enzymatic processes of modifying fats and oils by utilizing lipases from various origins [11]. Enzymatic conversion of fats has been reported by various researchers [12-14]. Ghazali et al. [14] have conducted in hexane media the enzymatic transesterification of palm olein with nonspecific and 1,3-specific lipases immobilized on Celite. The effects of transesterification of palm olein by the various lipases resulted in changes in the triglycerides mixture and no clear correlation between the enzyme positional specificity and the products formed was found.

Palm oil is extracted from the fruit of oil palm, *Elaeis guineensis*. It is one of the traditional fats that have been widely used throughout the world in the human diet. Global palm oil production was estimated to 45.9 millions of tons during 2009–2010, accounting for 40 % of the worldwide production of total dietary oils [15]. Palm oil contains a mixture of high and low melting points triacylglycerols. Using a simple dry fractionation process under controlled conditions, palm oil can be resolved into two fractions, namely olein (liquid fraction) and stearin (solid fraction) [16]. Palm olein is rich in low melting point triacylglycerols and is the mostly used fraction [8]. However, the high melting point fraction (palm stearin, melting point ranging from 45 to 55°C) is hardly used in manufacturing edible fats due to its low plasticity [16]. Nevertheless, palm stearin deserves attention as a potential hard fat of vegetable origin to replace hydrogenated lipids. It might be appropriately blended and interesterified with liquid oils in order to modify the physical characteristics of the mixture to meet the functional properties and the quality required for margarine preparation.

Lai et al. [17] have used nonspecific and 1,3-specific lipases to transesterify mixtures of palm stearin and sunflower oil at a 40:60 mass ratio in a solvent-free medium. The authors have found that the palm stearin and sunflower oil mixtures were converted to a more fluid product. In the same context, Lai et al. [10] have also transesterified a mixture of palm stearin and palm kernel olein using the same lipases. They reported that the enzymatic transesterification was able to produce fat mixtures with substantially lower melting points by repositioning the fatty acids of triglycerides in the higher melting range to form lower-or middle-melting components.

In the same context, this work reports the synthesis of a fatty phase by transesterification of palm stearin and palm olein using an immobilized *Rhizopus oryzae* lipase as a biocatalyst. The maximal rate of palm stearin that is usually added to a standard table margarine formulation is 10% [16]. The purpose of this work was to maximise the palm stearin proportion in the fatty phase (higher than 35%). A margarine was prepared out of the palm stearin/palm olein interesterified (40/60; w/w) mixture used as fatty phase. Slip melting point and rheological properties of the margarine were studied. In order to improve the colour, powder of dry bark orange was added to one margarine sample which rheological properties were studied.

## Methods

### Production and immobilization of lipase

*Rhizopus oryzae* lipase was produced as described by Ben Salah et al. [18]. The enzyme immobilization was made onto CaCO_3_ as described by Ghamgui et al. [19]. The activity of the immobilized lipase was measured titrimetrically with a pH-stat, under the standard assay conditions described previously by Rathelot et al. [20] using olive oil (10%) emulsion as substrate. One international unit (IU) of lipase activity was defined as the amount of lipase that catalyzes the liberation of 1 μmol of fatty acid per minute at pH 8.5 and 37 °C.

### Fractionation process

Refined, bleached and deodorized palm oil, of iodine value 50, was obtained from the Tunisian Olive Oil Office. It was fractionated in the laboratory by a dry fractionation process, according to the method described by Thiagarajah [21]. RBD palm oil was melted and kept homogenized at 70°C to destroy all crystals present. The melted oil was stirred at 25 rpm to keep it homogenized. The temperature was then decreased to 30°C. After stabilization, two fractions were obtained, a solid fraction: palm stearin (PS) and a liquid fraction: palm olein (PO). They were separated by vacuum filtration.

### Enzymatic transesterification

Transesterification reactions using various palm stearine/palm olein (PS/PO) mixtures (35/65, 40/60 and 60/40; w/w) were conducted in screw-capped flasks containing 10 g of total lipids. Reactions were monitored for 72 hours at 50 °C using 1000 IU of immobilized lipase and under stirring (200 rpm). The biocatalyst was removed from reaction samples by centrifugation at 8000 rpm for 5 min, washed thoroughly with hexane and reused in the reusability study and the supernatant was used for determining the melting point or the triacylglycerols composition. A control experiment was carried out in the same conditions without adding the enzyme.

### Slip melting point (SMP)

SMP was determined according to the AOCS Method Cc.3.25 [22]. Capillary tubes filled each with 1 cm high column of fat were chilled in a refrigerator at 4°C before being immersed in a beaker of cold distilled water. The water was stirred and heated and the temperature was recorded when the column of fat rises in the tube.

### Iodine value by Wijs method (IV) and acid value (AV)

The iodine value and the acid value were determined according to the AOCS Method Cd-25 and Cd 3a-63, respectively [22]. The reported values are means of three measurements.

### Triacylglycerols (TGs) profiles

The TGs profiles of the transesterified and non-transesterified blends of PS:PO were analyzed using a reversed-phase high performance liquid chromatography (HPLC, Shimadzu SCL-6A) equipped with a refractive index detector and with two C18 reverse phase analytical Shim-Pack CLC-ODS (M) columns connected in series for a good separation (the first column (15 cm x 4.6 mm) and the second (25 cm x 4.6 mm). During analysis, the column was maintained at 45 °C. The mobile phase was a mixture of acetone/ acetonitrile at a ratio of 70:30 (v/v) and at a flow rate of 1.5 mL/min. Identification of TGs was done by comparison of retention times with those of commercial TGs standards.

### Fatty acid analysis

Samples were dissolved in 0.5 mL of hexane. Then, 0.2 mL of potassium hydroxide in methanol (2 N) was added for the fatty acid methylation process. The mixture was vortexed then centrifuged and the upper phase containing fatty acid methyl esters were analyzed by Gas Chromatography (GC). GC analyses were performed on a Shimadzu, GC 17 A chromatograph, equipped with a flame ionization detector and a capillary column (50 m × 0.32 mm × 0.5 mm, PERICHROM Sarl, France). The oven temperature was programmed as follows: the initial temperature (100°C) was raised to 150°C at a rate of 30°C/min and held at this temperature for 5 min. The temperature was then increased to 190°C (at 10°C/min) and maintained for 14 min before being increased (at 5°C/min) to 255°C and held for 10 min. The injector and detector temperatures were 255 and 270°C, respectively. Nitrogen was the carrier gas with a flow rate of 1.13 mL/min. The identification of fatty acids was achieved by comparing retention times with those of authentic standards analysed under the same conditions. Peak areas were measured with an HP computing integrator. Results which are means of triplicates were expressed as w/w percentage of total fatty acids [23].

### Margarine formulation and preparation

Unless otherwise indicated, the composition of the prepared margarine was: 81% transesterified fat, 14.8% water, 1% hard boiled egg yolk used as emulsifier, 0.1% sugar, 0.1% salt and 3% butter. Hard boiled egg yolk and butter were dissolved in the heated oil phase (50°C), and sugar and salt were dissolved in the water phase. An Overhead Stirrer (Bioblock equipped with propeller stirrer) was used to homogenize the margarine samples. Both phases were stirred and cooled rapidly in order to obtain small and uniform crystals [24]. Three margarine samples were prepared using the transesterified fat at a PS/PO mass ratio of 40/60. One margarine sample contained no butter and 84% of fat. The second contained 3% of butter. The third sample contained 3% of butter and 0.2% of a powder of dry bark orange. A control margarine sample was prepared using non transesterified PS/PO mixture at a ratio of 40/60, w/w. Commercial margarine containing hydrogenated fat was also studied. It was purchased from a local supermarket in Sfax, Tunisia.

### Rheological analysis

For all margarine samples, viscosity was followed at10°C with a Stress Tech Rheologica Rheometer (Rheologica Instruments AB, Lund, Sweden) conducted with a steel cone-plate (C40/4).

## Results and discussion

### Fractionation of palm oil

Dry fractionation process was applied to separate palm oil into two fractions, olein and stearin, without the addition of chemicals or solvents. The dry fractionation is based on differences in melting points of triacylglycerols [25-27], and is a thermomechanical separation process where the high and low melting triacylglycerols are separated by partial crystallization, followed by filtration [28].

Table 1 summarizes the physicochemical characteristics of palm oil and its fractions. During fractionation, triacylglycerols are redistributed into two phases. As fractionation proceeds, the more saturated triacylglycerols are gradually concentrated in the solid phase (stearin) and the more unsaturated one is left in the liquid phase (olein). Major saturated fatty acids were lauric (C12:0), myristic (C14:0), palmitic (C16:0) and stearic acids (C18:0) whereas major unsaturated fatty acids consisted of three major ones: oleic (C18:1), linoleic (C18:2), and linolenic (C18:3) acids.

**Table 1:** Characteristics and fatty acids composition of palm fraction; SFA: saturated fatty acids; UFA: unsaturated fatty acids

**Sample**	**Characteristics**			**Fatty acids composition (%)**								
**Palm fraction**	**Saponification value (mgKOH/g)**	**Iodine value (gI**_**2**_**/100 g)**	**Slip melting point (°C)**	**C 12:0**	**C 14:0**	**C 16:0**	**C 18:0**	**C 18:1**	**C 18:2**	**C 18:3**	**∑ SFA**	**∑ UFA**
**Palm oil**	200	50	42	0.15	0.90	43.30	4.00	41.82	9.60	0.24	48.35	51.66
**Palm stearin**	199	38	54	0.10	1.08	55.94	3.73	32.40	6.65	0.10	60.85	39.15
**Palm olein**	201	56.4	28	0.10	0.45	41.38	4.34	43.28	10.25	0.20	46.27	53.73

Iodine value (IV) reflects the unsaturation level of fats and oils. Among oil palm fractions, palm olein had a higher IV and lower slip melting point as compared to stearin. This is in agreement with its higher content in unsaturated fatty acids.

### Changes in slip melting points during transesterification

Transesterification is used to modify the properties of triacylglycerol mixtures. The fatty acid chains are redistributed within the triacylglycerol molecules resulting generally in a change in the melting characteristics of the product in comparison with the starting mixture [9].

In a preliminary study (data not shown), we have checked the transesterification performance of *Staphylococcus xylosus* and *Rhizopus oryzae* lipases produced in our laboratory [18,29] and immobilized onto CaCO_3_[19] and Chirazyme® L-9 a commercial immobilized lipase from *Rhizomucor miehei*. These enzymes were tested in the transesterification of a 50:50 PS/PO (w,w) mixture. Upon a reaction time of 24 h a significant shift in the SMP was obtained for the ROL- and Chirazyme -catalyzed reactions (from 44 to 40-41°C). However, no change in SMP was observed for *S. xylosus-* lipase*-*catalyzed reaction. These results are presumably related to the specificity of the ROL toward long-chain triacylglycerols [19] as compared to *S. xylosus* lipase more active on short-chain triacylglycerols [29], since PS/PO mixture is rich in long chain fatty acids.

In addition, CaCO_3_ was used as a support to immobilize ROL by adsorption. The choice of this support was based on results reported by Ghamgui et al. [19] which have tested five supports (Silica gel 60, AmberliteIRC-50, Carboxy-methyl Sephadex, Celite 545 and CaCO_3_) to immobilize ROL by the adsorption technique. The authors have found that CaCO_3_ was the most suitable support to immobilize ROL since they have obtained a high yield of immobilization (93.75 %).

Moreover, the most widely used lipases in the synthesis reaction were commercial lipases which are usually microbial extracellular enzymes produced by fermentation of yeasts, fungi or bacteria. Unfortunately, the utilization of commercial enzymes to perform the transesterification is still very expensive. The use of low-cost lipases like *Rhizopus oryzae* lipase (ROL) may increase the process economical and environmental attractiveness. No previous studies involving ROL in the transesterification reaction to produce fat phase suitable for margarine preparation were reported.

**Figure 1:**
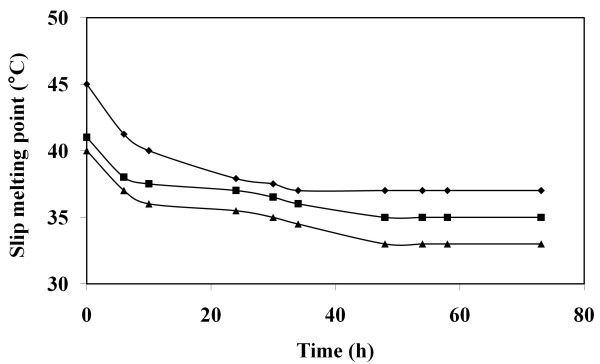
** Slip melting point of palm stearin (PS)/ palm olein (PO) mixture during transesterification.** PS/PO (w/w): 35/65 (closed triangles) 40/60 (closed squares) 60/40 (closed diamonds). Reaction conditions: 1000 IU ROLi, 50 °C and 200 rpm.

The slip melting points (SMP) of three blends containing various palm stearin (PS)/ palm olein (PO) ratios (35/65, 40/60 and 60/40; w/w) decreased during transesterification time course (Figure 1). This is likely to be explained by the rearrangement of fatty acids within triacylglycerols (TGs) using ROL as a biocatalyst. No changes were observed in the SMP for control experiments carried out without enzyme. After 48 h of reaction time, the SMP remained constant. This might indicate that the reaction reached the equilibrium. These results are in agreement with the decrease in the SMP of palm stearin/palm kernel olein mixture after transesterification using *Pseudomonas* lipase [10]. The SMP increases as the amounts of PS augment in the starting blends (Figure 1), due to a greater content in high-melting triacylglycerols in PS.

SMP of PS/PO (40/60; w/w) decreased after 24 h of reaction time from 41 °C to 37 °C. This suggests a possible usage of this blend in the preparation of a table margarine for which a slip melting point around body temperature is required for a proper mouthfeel. Furthermore, stopping the reaction at 24 h would allow to reduce the reaction cost. This blend was subjected to further analysis in order to be used in margarine formulation.

To check the quality of the blend at the end of the reaction, the acid value was determined. It is defined by Woodlat [30] as the number of mg of KOH required to neutralize 1 g of fatty acids in an oil. As shown in Table 2, the acid value increased slightly from the starting blend (from 0.14 to 0.39 mg KOH/g oil). Such value is still acceptable and there is no need to further refine the final product.

**Table 2:** **Triacylglycerol composition (%) and acid values (AV) (mg KOH/g oil) of palm stearin-palm olein blend (40:60, w/w) before and after 24 h of transesterification by with* R. oryzae* lipase (fatty acids: P, Palmitic acid; O, oleic acid; L, linoleic acid; Ln, Linolenic acid and S, stearic acid)**

**Sample**	**AV**	**Triacylglycerols**							
		**LnLnO**	**LOO/LnOO**	**POL**	**OOO**	**POP/POO**	**PPP**	**SOO**	**POS**
**Non transesterified blend**	0.14	1.2	5.56	2.11	8.46	37	33	1.33	4.24
**Transesterified blend**	0.39	5.7	8	2.3	12.02	30.1	26.79	1.1	5.33

### Changes in triacylglycerol profiles during transesterification

**Figure 2:**
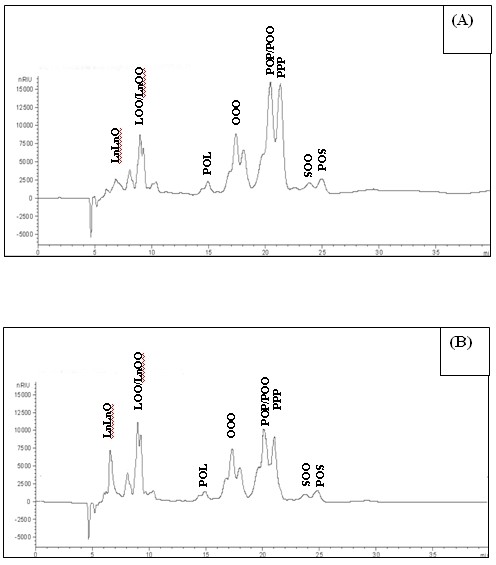
HPLC Chromatograms of (A) non-transesterified and (B) transesterified palm stearin and palm olein blend (40:60) obtained after 24 h of reaction time (P, Palmitic; O, oleic acid; L, linoleic; Ln, Linolenic acid; S, stearic acid).

PS/PO (40/60; w/w) mixture was analyzed by HPLC to determine the triacylglycerols profile before (Figure 2A) and after 24 h of enzymatic transesterification (Figure 2B). Prior to transesterification, the blends contained higher proportions of high melting point triacylglycerols, such as POP (37%), POS (4.24%), and PPP (33%). After transesterification, the amounts of these saturated TGs decreased and lower melting point TGs such as LOO (8%), LnLnO (5.7%), and OOO (12.02%) were formed (Table 2). The changes in the triacylglycerols composition were due to the rearrangement of the fatty acids during interesterification.

These results are in agreement with previous findings [31-34] that transesterification of fats and oil blends was often accompanied by changes in the SMP.

### Preparation of margarines and rheological analysis

**Figure 3:**
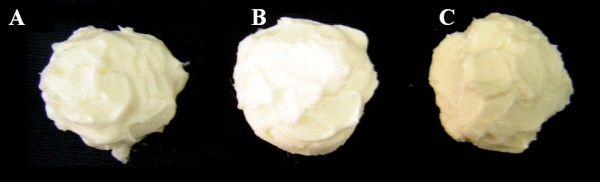
** Margarine prepared with PS:PO (40:60) blend. (A) **non transesterified blend, **(B)** transesterified blend, **(C)** transesterified blend containing powder of dry bark orange.

The PS/PO (40/60; w/w) blend transesterified with 1000 IU of ROLi for 24 h of reaction time was used as a fatty phase to produce table margarine samples which were prepared in compliance with the margarine formulation recommendations [35]. Hard boiled egg yolk (1%) was used as emulsifier. Egg yolk contains about 10% of phospholipids which are desirable emulsifiers widely used in food formulations [36]. Egg yolk was boiled in order to avoid any risk of product contamination. Figure 3 shows three margarine samples. The first was prepared with non transesterified blend (Figure 3A) and the two others were prepared with transesterified blend (Figure 3B and C). In order to give the product a butter-like attractive colour, powder of dry bark orange was added to the last margarine preparation (Figure 3C). This powder used as a source of carotenes gave to the margarine sample a yellow-orange colour, very close to that of butter. A patent describing this formulation has been filed with the patent office [37].

**Figure 4:**
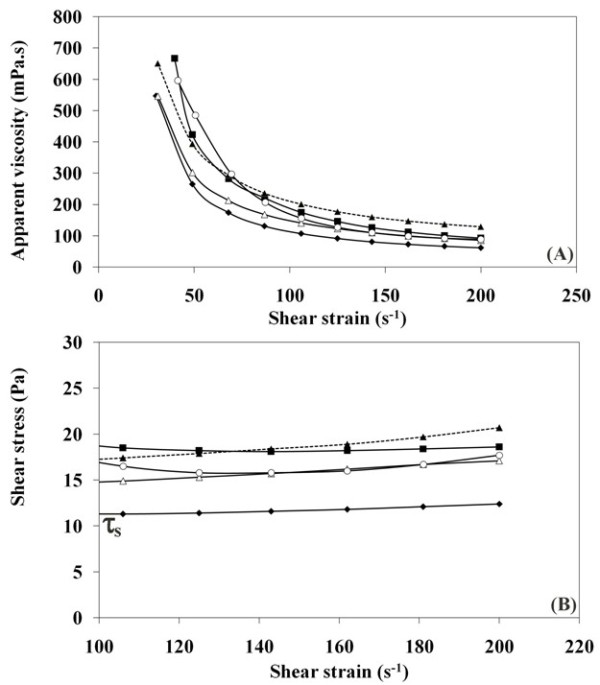
** Rheological data, (A) Effect of shear strain rate on the apparent viscosity.** (**B**) Effect of shear strain rate on the shear stress. Margarines prepared with PS:PO (40:60) blend. (closed diamonds) non transesterified blend added 3 % butter; (closed squares) transesterified blend; (closed triangles) transesterified blend added 3 % butter; (open triangles) transesterified blend added 3 % butter and 0.2 % powder of dry bark orange; (open circles) commercial margarine sample.

Figure 4A shows the variations of margarine apparent viscosity when increasing the shear strain rate. As expected, the viscosity declines strongly when shear strain rate increases for all studied margarines. They are characterized by a non-Newtonian rheological behaviour. Margarines prepared with transesterified fat blends showed a rheological behaviour comparable to that of the commercial one. Margarine prepared with the non transesterified blend displayed the greatest hardness since it exhibited the lowest variation of apparent viscosity when the shear strain rate increased from 50 s^-1^ to 200 s^-1^. This rheological behaviour is likely to be the consequence of the high slip melting point of the non transesterified blend.

Figure 4B shows the variations of shear stress with increasing the shear strain rate. As the shear strain rate was increased, no significant deformation took place until the resulting stress reaches the shear yield stress value (τ_s_). The fat behaves like a rigid solid until the shear stress exceeds the limit value (τ_s_), and the fat starts flowing like a Newtonian liquid. This curve is a characteristic of the plastic fat behaviour of margarine [38]. This behaviour is due to the presence of a fat crystal network [39]. Triacylglycerol crystals of margarine fatty phase are associated with each other by means of primary and secondary bonds [39], leading to a three-dimensional structure that maintains the solid state.

Table margarine must be spreadable when taken straight from the refrigerator. That’s why all rheological analysis were performed at 10°C; temperature of refrigerator’s butter compartment. τ_s_ which represents also a measure of margarine spreadability was determined. Margarines prepared with transesterified fat had a better spreadability than those prepared with a non transesterified blend. Furthermore, the spreadability of transesterified fat margarine was similar to that of a commercial product. Since butter might be added to a margarine preparation at a maximal rate of 3% [35], we checked that adding 3% of butter to the zero trans fat margarine did not affect its rheological behaviour. The rheological behaviour of the margarine prepared with powder of dry bark orange was fairly similar to the commercial product.

### Reusability of the biocatalyst

**Figure 5:**
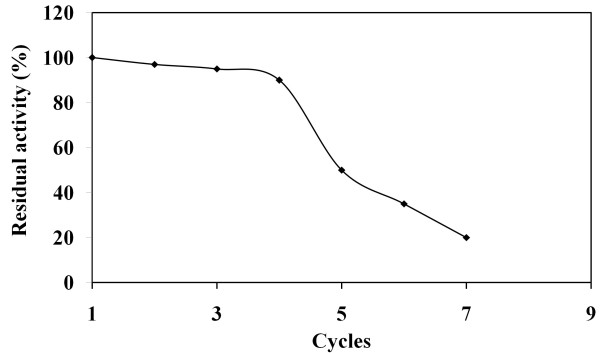
** Effect of repeated use of biocatalyst.** Reaction conditions: an enzyme amount of 1000 IU, a PS/PO mass ratio of 40/60 and an incubation time of 24 h.

One important factor limiting the use of lipase catalyzed reactions at an industrial scale is the high enzyme cost which can determine the economic viability of any biosynthetic process [40]. The cost efficiency of the reaction could be greatly improved by reusing the lipase for several reactions [41]. After 24 h of reaction time, the same immobilized enzyme was reused many times. After a reaction cycle, the immobilized lipase was separated from the reaction mixture by centrifugation, washed thoroughly with hexane and a fresh substrate sample was added to the same enzyme. No significant decrease in the enzyme activity was observed for 4 cycles of reuse and the residual activity was 90%. Upon the fifth cycle, the enzyme lost 50% of its initial activity (Figure 5). The reason for the reduction of enzyme activity can be partly explained by desorption of lipase from the adsorbent or denaturation after many cycles of use. These results indicate the feasibility of enzyme recycling in this system. We used in a previous study [42] the same biocatalyst for 20 cycles in the synthesis of wax esters without significant loss of its activity. This difference between the two reactions could be explained by the composition of the reaction medium (solvent, water content, substrate chemistry) or the operating conditions.

## Conclusion

This study has shown that enzymatic transesterification was an effective way to modify the physical and chemical properties of palm stearin and palm olein blends. The enzymatic transesterification allows to obtain fats with optimum melting characteristics for use in margarine production. The rheological analysis showed that margarine prepared with the transesterified blend showed a better spreadability than that of a control margarine prepared with non transesterified fat. Adding powder of dry bark orange to margarine preparation improved its colour and fairly affected its spreadability and rheological behaviour.

## Competing interests

The authors declare that they have no competing interests.

## Authors’ contributions

MS, HG and FF designed the experiments, carried out the synthesis and the analysis of margarine and drafted the manuscript.YG and NM have conceived research and approaches and have given final approval of the version to be published. All authors read and approved the final manuscript.

## References

[B1] Chrysam MM Margarines and spreads 1996 New York: John Wiley and Sons

[B2] List GR Emken EA Kwolek WF Simpson TD Dutton HJ Zero trans margarines: preparation, structure, and properties of interesterified soybean oil-soy trisaturate blends J Am Oil Chem Soc 1977 54 408 413 10.1007/BF02671021

[B3] Fomuso LB Akoh CC Enzymatic modification of high-laurate canola to produce margarine fat J Agric Food Chem 2001 49 4482 4487 10.1021/jf010444u 11559158

[B4] Mensink RP Katan MB Effect of dietary trans-fatty acids on high-density and low-density lipoprotein cholesterol levels in healthy subjects N Engl J Med 1990 23 439 445 2374566 10.1056/NEJM199008163230703

[B5] Zock PL Katan MB Hydrogenation alternatives: effects of trans-fatty acids and stearic acid versus linoleic acid on serum lipids and lipoproteins in humans J Lipid Res 1992 33 399 410 1569387

[B6] Willett WC Stampfer MJ Manson JE Colditz GA Speizer FE Ross MB Sampson LA Hennekens CH Intake of trans-fatty acids and risk of coronary heart disease among women Lancet 1993 341 581 585 10.1016/0140-6736(93)90350-P 8094827

[B7] Micha R Mozaffarian D Trans fatty acids: effects on cardiometabolic health and implications for policy Prostaglandins Leukot Essent Fatty Acids 2008 79 147 152 10.1016/j.plefa.2008.09.008 18996687 PMC2639783

[B8] Norizzah AR Chong CL Cheow CS Zaliha O Effects of chemical interesterification on physicochemical properties of palm stearin and palm kernel olein blends Food Chem 2004 86 229 235 10.1016/j.foodchem.2003.09.030

[B9] Berben PH Groen C Christensen MW Holm HC Interesterification with immobilized enzymes Society of Chemical Industry 2000 121 1 2

[B10] Lai MO Ghazali HM Let CC Effect of enzymatic transesterification on the fluidity of palm stearin-palm kernel olein mixtures Food Chem 1998 63 155 159 10.1016/S0308-8146(98)00046-6

[B11] Reshma MV Saritha SS Balachandran C Arumughan C Lipase catalyzed interesterification of palm stearin and rice bran oil blends for preparation of zero trans shortening with bioactive phytochemicals Bioresour Technol 2008 99 5011 5019 10.1016/j.biortech.2007.09.009 17949974

[B12] Husum TL Pederson LS Nielson PM Christensen MW Kristensen D Holm HC Enzymatic interesterification: process advantages and product benefits Palm Oil Development 2004 39 7 10

[B13] Ronne TH Yang T Mu H Jacobsen C Xu X Enzymatic interesterification of butter fat with rapeseed oil in a continuous packed bed reactor J Agric Food Chem 2005 53 5617 5624 10.1021/jf050646g 15998124

[B14] Ghazali HM Hamidah S Che Man YB Enzymatic transesterification of palm olein with nonspecific and 1,3-Specific lipases J Am Oil Chem Soc 1995 72 6 633 639 10.1007/BF02635647

[B15] FAOSTAT Online Statistical Service 2012 http://faostat.fao.org.

[B16] Lai OM Ghazali HM Cho F Chong CL Physical and textural properties of an experimental table margarine prepared from lipase-catalysed transesterified palm stearin:palm kernel olein mixture during storage Food Chem 2000 71 173 179 10.1016/S0308-8146(00)00084-4

[B17] Lai OM Ghazali HM Chong CL Use of enzymatic transesterified palm stearin-sunflower oil blends in the preparation of table margarine formulation Food Chem 1999 64 83 88 10.1016/S0308-8146(98)00083-1

[B18] Ben Salah A Fendri K Gargouri Y La lipase de Rhizopus oryzae: production, purification et caractéristiques biochimiques Revue Française des Corps Gras 1994 4 133 137

[B19] Ghamgui H Karra-Châabouni M Gargouri Y 1-Butyl oleate synthesis by immobilised lipase from Rhizopus oryzae: a comparative study between n-hexane and solvent-free system Enzyme Microb Technol 2004 35 355 363 10.1016/j.enzmictec.2004.06.002

[B20] Rathelot J Julien R Canioni P Coereli C Sarda L Studies on the effect of bile salt and colipase on enzymatic lipolysis Improved method for the determination of pancreatic lipase and colipase. Biochimie 1975 57 1117 1122 10.1016/s0300-9084(76)80572-x 1222120

[B21] Thiagarajah T Refining of palm and palm kernel oils 1992 Malaysia: Selected readings on palm oil for participants of palm oil familiarization programme. PORIM. Ministry of Primary Industries

[B22] AOCS Official and tentative methods of the American Oil Chemists’ Society; Champaign 1990 Champaign.IL: AOCS Press

[B23] Sagdiç O Donmez M Demirci M Comparison of characteristics and fatty acid profiles of traditional Turkish yayik butters produced from goats, ewes or cows milk Food Control 2004 15 485 490 10.1016/j.foodcont.2003.07.003

[B24] Che Man YB Swe PZ Thermal analysis of failed batch palm oil by differential scanning calorimetry J Am Oil Chem Soc 1995 72 1529 1532 10.1007/BF02577848

[B25] Ng WL Nucleation from palm oil melt; PORIM 1989 Malaysia: Ministry of Primary Industries

[B26] Siew WL Ng WL Diglycerides content and composition as indicators of palm oil quality J Sci Food Agric 1995 69 73 79 10.1002/jsfa.2740690112

[B27] Siew WL Ng WL Effect of diglycerides on the crystallization of palm olein J Sci Food Agric 1996 71 496 500 10.1002/(SICI)1097-0010(199608)71:4<496::AID-JSFA616>3.0.CO;2-W

[B28] Kellens M New developments in the fractionation of palm oil; PORIM 1993 Malaysia: Ministry of Primary Industries

[B29] Mosbah H Sayari A Verger R Gargouri Y Gly311 residue triggers the enantioselectivity of Staphylococcus xylosus lipase: a monolayer study J Colloid Interface Sci 2007 310 196 204 10.1016/j.jcis.2007.01.073 17335837

[B30] Woodlat EE The manufacture of soap, other detergents and glycerine 1985 2 England: Ellis Harwood Pub

[B31] Forssell P Kervinen P Lappi M Linko P Suortti T Poutanen K Effect of enzymatic interesterification on the melting point of tallow-rapeseed oil (LEAR) mixture J Am Oil Chem Soc 1992 69 126 129 10.1007/BF02540561

[B32] Foglia TA Petruso K Feairheller SH Enzymatic interesterification of tallow-sunflower oil mixtures J Am Oil Chem Soc 1993 70 281 285 10.1007/BF02545309

[B33] Ghazali HM Maisarah A Yusoff S Yusoff MSAM Triglyceride profiles and melting properties of lipase catalysed transesterified palm stearin and coconut oil Asia Pac J Mol Biol Biotechnol 1995 3 280 289

[B34] Zainal Z Yusoff MSAM Enzymatic Interesterification of palm stearin and palm kernel olein J Am Oil Chem Soc 1999 76 1003 1008

[B35] Codex alimentarius Codex standard for margarine Codex Stan 32 1981 2001 8 1 4

[B36] Luz EP Tong W Egg-yolk lipid fractionation and lecithin characterization J Am Oil Chem Soc 2005 82 571 578 10.1007/s11746-005-1111-4

[B37] Sellami M Ghamgui H Frikha F Gargouri Y Miled N Production de margarine végétale dépourvue d’acides gras trans.INNORPI. Tunisian patent filing number 2010–0311 2010

[B38] Segura JA Herrera ML Anon MC Margarines: a rheological study J Am Oil Chem Soc 1995 72 375 378 10.1007/BF02541099

[B39] Haighton AJ Work softening of margarine and shortening J Am Oil Chem Soc 1965 42 27 30 10.1007/BF02558248 14228466

[B40] Krishna SH Sattur AP Karanth NG Lipase-catalyzed synthesis of isoamyl isobutyrate optimization using central composite rotatable design Process Biochem 2001 37 9 16 10.1016/S0032-9592(01)00161-3

[B41] Compton DL Laszlo JA Berhow MA Lipase-catalyzed synthesis of ferulate esters J Am Oil Chem Soc 2000 77 513 519 10.1007/s11746-000-0082-9

[B42] Aissa I Sellami M Kamoun A Gargouri Y Miled N Optimization of immobilized lipase-catalyzed synthesis of wax esters by response surface methodology Curr Chem Biol 2012 6 77 85 10.2174/187231312799984376

